# Recurrent Vulvovaginitis as an Unusual Presentation of Diagnosed Obstructed Hemivagina and Ipsilateral Renal Anomalies (OHVIRA) Syndrome in an Adolescent: A Case Report and Review of the Literature

**DOI:** 10.3390/jcm15020440

**Published:** 2026-01-06

**Authors:** Ju Seok Yang, Ji Eun Park, Hyen Chul Jo

**Affiliations:** 1 Department of Obstetrics and Gynecology, Gyeongsang National University Changwon Hospital, Changwon 51427, Republic of Korea; 2 Department of Obstetrics and Gynecology, School of Medicine, Gyeongsang National University, Jinju 52727, Republic of Korea; 3 Institute of Health Science, Gyeongsang National University, Jinju 52828, Republic of Korea

**Keywords:** obstructed hemivagina and ipsilateral renal anomalies (OHVIRA) syndrome, vulvovaginitis, transverse vaginal septum, adolescence

## Abstract

**Background:** Vulvovaginitis is a common condition in pediatric and adolescent female patients and is most frequently caused by infection. Although non-infectious etiologies are less common, they should be considered, particularly in cases that are refractory to standard treatment. **Case:** We report a rare case of a 16-year-old adolescent who was ultimately diagnosed with obstructed hemivagina and ipsilateral renal anomalies (OHVIRA) syndrome after experiencing recurrent vulvovaginitis for more than one year. Despite repeated antimicrobial treatments, her symptoms persisted. Further imaging studies revealed uterine didelphys with an obstructed hemivagina and ipsilateral renal agenesis. Surgical resection of the vaginal septum resulted in complete resolution of symptoms. **Conclusions:** Müllerian anomalies, such as OHVIRA syndrome, should be considered in the differential diagnosis for adolescents with recurrent vulvovaginitis refractory to conventional therapy. Delayed diagnosis may result in complications that significantly impair both quality of life and future reproductive potential.

## 1. Introduction

Vulvovaginitis is defined as inflammation of the vulva and vagina and is a common condition in pediatric and adolescent female patients. Approximately 75% of women experience vulvovaginitis at least once during their lifetime, with many experiencing their first episode during adolescence [1]. The etiology, clinical manifestations, and management strategies vary depending on patient’s age. In adolescent population, vulvovaginitis can be broadly categorized into infectious and non-infectious causes. Infectious etiologies are more prevalent and include bacterial vaginosis, vulvovaginal candidiasis, and Trichomonas vaginalis infection [2]. Among these, bacterial vaginosis is the most common, affecting approximately 23% of adolescents [3]. Non-infectious causes are regarded as important in adolescent vulvovaginitis; these include vulvar inflammation and irritation resulting from chemical irritants, retained foreign bodies, trauma, poor hygiene, and Müllerian anomalies associated with partial outflow tract obstruction.

Obstructed hemivagina and ipsilateral renal anomalies (OHVIRA) syndrome is a rare congenital disorder classified as an obstructive Müllerian anomaly. It is characterized by a triad of uterine duplication (didelphys, bicornuate, or septate uterus), obstructed hemivagina, and ipsilateral renal agenesis or other renal anomalies. The most common clinical manifestations include dysmenorrhea, abdominal or pelvic pain, abnormal vaginal bleeding, pelvic masses, menstrual irregularities, and urological symptoms [4].

Here, we report a rare case of OHVIRA syndrome in a 16-year-old adolescent who presented with recurrent vulvovaginitis persisting for more than one year, representing an unusual clinical presentation of this condition.

## 2. Case Report

A 16-year-old sexually inactive female presented to the gynecology outpatient clinic with persistent purulent vaginal discharge and recurrent episodes of vulvovaginitis over a one-year period. The discharge was yellowish and malodorous. She had previously received treatment at external medical facilities; however, her symptoms repeatedly recurred despite therapy.

Menarche had occurred at 12 years of age, with regular 30-day menstrual cycles. The patient reported no significant dysmenorrhea requiring medication. Her last menstrual period was one week prior to the initial visit. There was no remarkable past medical or surgical history.

Upon physical examination, secondary sexual characteristics were appropriate for age, consistent with Tanner stage III breast development and pubic hair. The vulva appeared hyperemic and excoriated, indicative of inflammation, and yellowish vaginal discharge was observed. Diagnostic evaluation included routine vaginal cultures and a multiplex PCR panel of 12 types of sexually transmitted infections (STIs). All STI tests were negative; however, the routine vaginal culture was positive for *Escherichia coli*. The patient was subsequently treated with targeted antibiotic therapy for one week.

**Figure 1:**
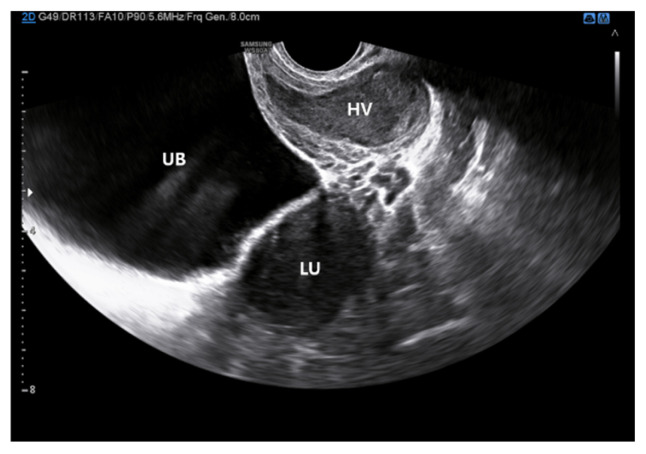
Preoperative transrectal ultrasound image demonstrates the uterus with the obstructed hemivagina of heterogeneous contents. (UB; urinary bladder, LU; uterus, HV; hemivagina).

**Figure 2:**
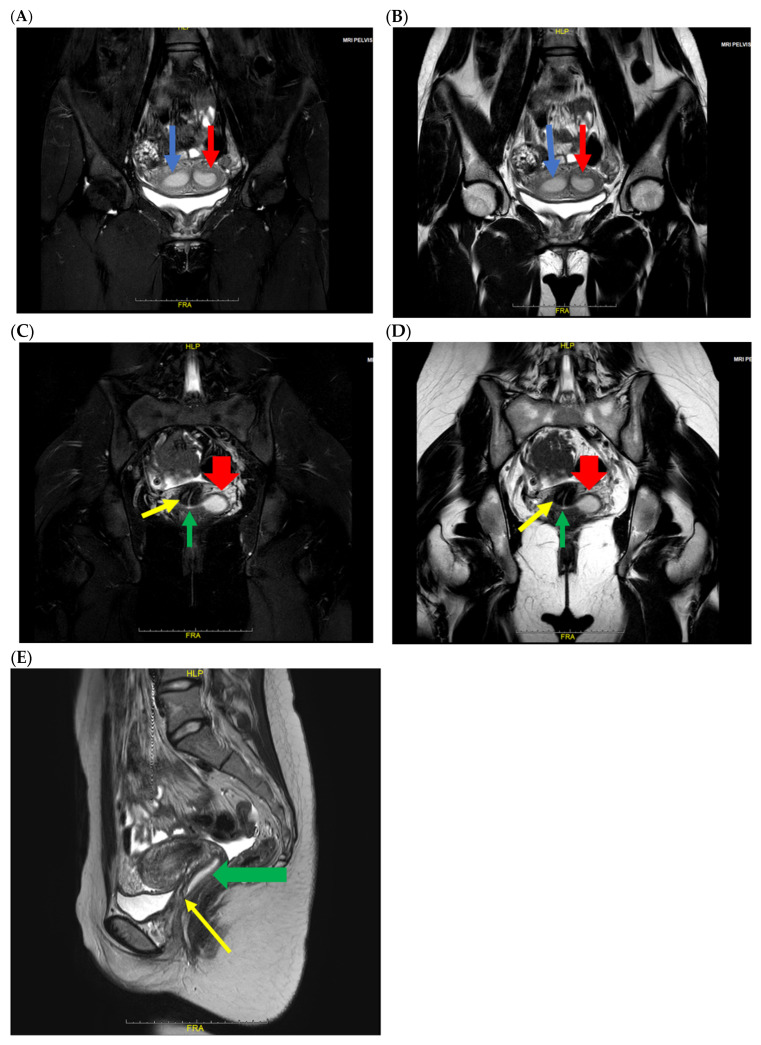
Coronal fat-saturated T2-weighted (**A**) and coronal T2-weighted (**B**) MRI images show two uterine cavities (blue arrow: right uterine cavity; red arrow: left uterine cavity). Coronal fat-saturated T2-weighted (**C**) and coronal T2-weighted (**D**) MRI images show two uterine cervices. The cavity is filled with hyperintense contents, which are connected to the left side of the uterine cervix (yellow arrow: right uterine cervix; green arrow: left uterine cervix; red arrow: distended hemivagina). Sagittal T2-weighted (**E**) MRI shows dilatation of the upper vagina and a vaginal septum (yellow arrow: transverse vaginal septum; green arrow: distended hemivagina).

Gynecologic examination ruled out an imperforate hymen. Transrectal ultrasonography identified a mixed-echogenic lesion inferior to the uterus without evidence of intrauterine fluid collection (Figure 1). Pelvic magnetic resonance imaging (MRI) confirmed the presence of uterine didelphys, characterized by two separate uterine cavities and two distinct cervices (Figure 2A,B). The upper vagina was distended and filled with heterogeneous T2 hyperintense contents (Figure 2C,D). Sagittal images demonstrated uterine didelphys associated with an upper transverse vaginal septum (Figure 2E), while the mid and distal vagina appeared normal.

**Figure 3:**
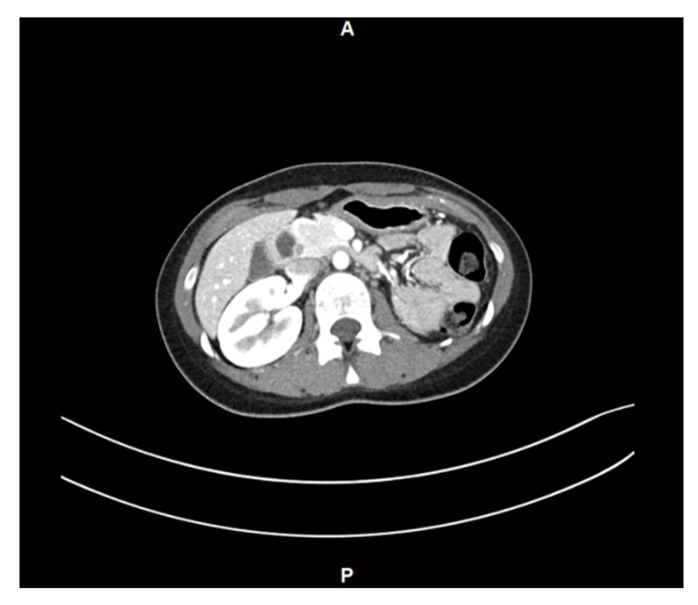
Contrast-enhanced computed tomography (CT) scan of abdomen shows the absence of left kidney.

Contrast-enhanced abdominal computed tomography performed to evaluate associated anomalies revealed left renal agenesis (Figure 3). Based on these findings, the patient was diagnosed with OHVIRA syndrome.

**Figure 4:**
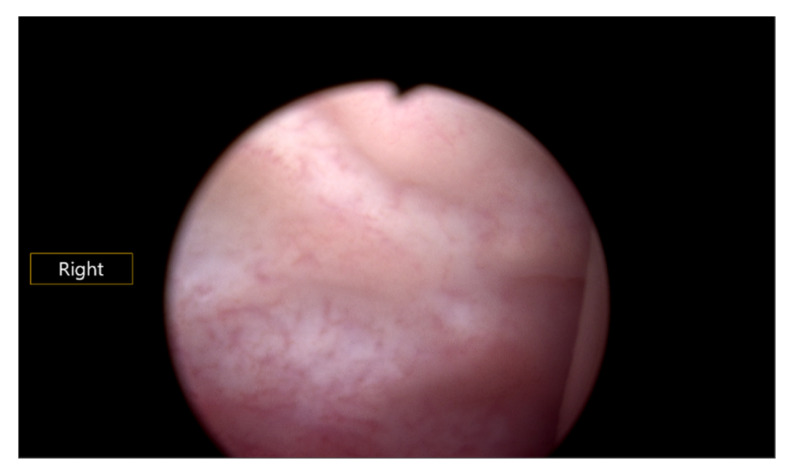
Hysteroscopic finding reveal the area of vaginal septum. The penetration of the vaginal septum is not visible.

Surgical intervention was performed under general anesthesia in the lithotomy position. Vaginoscopic hysteroscopy was initially employed to localize the vaginal septum. Because no visible opening was identified (Figure 4), the septum was localized by gentle pressure and observation of purulent discharge. Following an unsuccessful attempt at hysteroscopic septoplasty, a wide excision of the septum was performed using elliptical forceps via a small-sized speculum. The left cervical os was identified beneath the septum. To ensure hemostasis and prevent postoperative adhesion, circumferential suturing was performed using 3-0 braided polyglactin 910 sutures (Vicryl; Ethicon, Somerville, NJ, USA).

**Figure 5:**
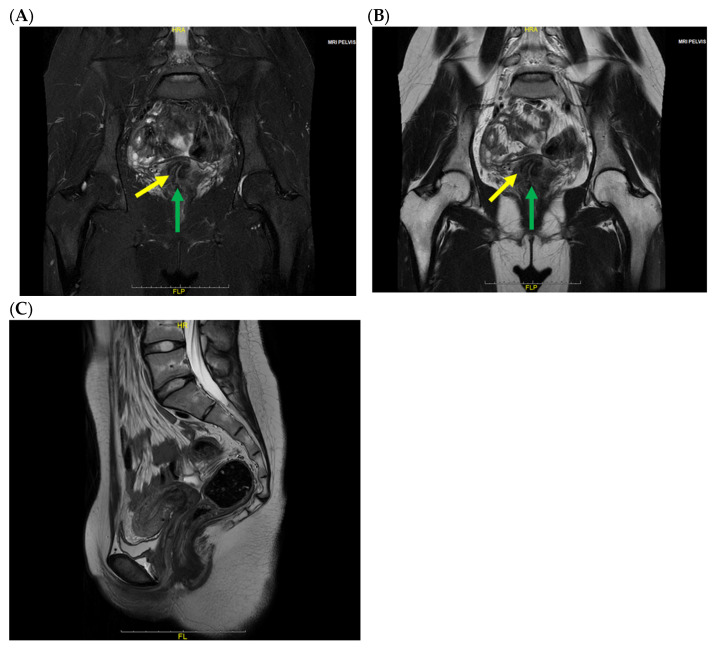
Coronal fat-saturated T2-weighted (**A**), Coronal T2-weighted (**B**) and Sagittal T1 weighted (**C**) MRI one year after surgery show no cavity which connected to the left side of uterine cervix and no obstructed hemivagina and vaginal septum. (Yellow arrow, right uterine cervix; green arrow, left uterine cervix).

The postoperative course was unremarkable, and the patient was discharged on postoperative day (POD) 3. At the one-year follow-up, pelvic MRI demonstrated no evidence of recurrent obstruction or vaginal stricture (Figure 5). The patient remained asymptomatic, with complete resolution of vulvovaginitis.

## 3. Discussion

OHVIRA syndrome, also known as Herlyn–Werner–Wunderlich syndrome, was first described by Purslow in 1922 and is a rare condition, accounting for approximately 0.1–3.8% of Müllerian duct anomalies [5]. It consists of a triad of uterine duplication, obstructed hemivagina, and ipsilateral renal anomalies. These malformations are a result of an anomalous development of Müllerian ducts around the eighth week of gestation. At 4 to 6 weeks of gestation, the fetal urogenital system begins to develop. At this stage, both male and female embryos possess Wolffian ducts and Müllerian ducts. Between 6 and 9 weeks of gestation, the Müllerian ducts develop while the Wolffian ducts regress under the influence of estrogen and in the absence of testosterone and the anti-Müllerian hormone (AMH) in female embryos. The Müllerian ducts initially form separately on each side of the uterus and gradually move toward the midline, where they fuse. By 9 to 12 weeks of gestation, the fused portion forms the uterovaginal canal that subsequently gives rise to the uterus, uterine cervix, and the upper two-third section of the vagina. Following fusion, the septum formed at the fusion line should disappear to establish a normal uterine cavity. It is believed that any disruption in this process can lead to the development of Müllerian anomalies. In the OHVIRA syndrome, failure in the fusion stage leads to the formation of uterine didelphys, and failure in the regression stage results in the development of a vaginal septum.

The clinical presentation of the OHVIRA syndrome varies depending on the specific anatomical abnormalities and the timing of onset. Patients may present with a diverse range of symptoms, including dysmenorrhea, abdominal or pelvic pain, pelvic masses, and menstrual irregularities. Urological symptoms such as urinary retention or incontinence may occur. While many patients presented asymptomatic, the most frequently reported symptoms were dysmenorrhea, abdominal pain, uterine bleeding, vaginal discharge, and urinary problem, in descending order of frequency [4]. Other reports also noted that dysmenorrhea and irregular vaginal bleeding are common symptoms in this condition [6]. It is rare for a patient to be diagnosed with recurrent vulvovaginitis, as in our case study. In the present case, this symptom can be attributed to the gradual accumulation of menstrual fluid caused by the vaginal septum, leading to the formation of hematocolpos that became infected and formed pyocolpos in the left hemivagina. This pus-like discharge then leaked through the opening of the penetration. Alternatively, the development of a pyocolpos may be facilitated by a small fistula between the duplicated vaginas. In such instances, an infection origination in the patent vagina can spread to the contralateral side via the fistula, leading to a pyocolpos and chronic vaginal discharge [7]. The clinical manifestations are largely determined by anatomical location and the degree of fenestration of the vaginal septum. Transverse vaginal septum is reported in approximately 1 in 2100 and 1 in 72,000 females [8]. Vaginal septa are located in 6% to 46% of the upper vaginal area, 22% to 40% of the middle section, and 15% to 72% of the lower vagina [9]. Typically, these septa are generally less than 1 cm in thickness and may feature a small central or eccentric perforation.

While prenatal diagnosis of this syndrome is rare, it is typically identified post-menarche, with a mean age at diagnosis of approximately 14 years. Early diagnosis and treatment are essential to prevent long-term complications and ensure favorable outcomes. Diagnostic delays, which can arise from various factors, may lead to serious sequelae such as pelvic inflammatory disease (PID) and endometriosis, ultimately compromising long-term fertility and quality of life. One study identified 17 patients who were diagnosed with this syndrome among those presenting of infertility as their primary symptom. The mean age at diagnosis was 23 years—significantly older than the typical age of diagnosis—suggesting a strong association between delayed diagnosis and impaired fertility. Following appropriate surgical intervention, five out of seven patients in that study successfully achieved pregnancy and delivered living infants [10]. These findings underscore that early detection and timely surgical management not only alleviate clinical symptoms but also facilitate favorable obstetric outcomes. Furthermore, a comprehensive evaluation for associated anomalies is imperative. Beyond common urogenital malformations—such as ipsilateral renal agenesis, multicystic dysplastic kidney, and compensatory contralateral renal hypertrophy—concomitant malformations have been documented in the skeletal (15.1%) and cardiovascular (0.4%) systems [6].

Ultrasonography and MRI are the primary modalities for diagnosing OHVIRA syndrome. Ultrasonography is typically the initial imaging tool of choice due to its accessibility and non-invasive nature. Common diagnostic findings included hematometra and hematocolpos; however, advanced cases may manifest with complications such as hematosalpinx/piosalpinge, tubo-ovarian abscess, and pyometra. MRI remains the gold standard to definitive diagnose, providing a precise assessment of uterine morphology and accurately delineating the site and extent of vaginal obstruction. Nevertheless, some studies suggest that while MRI is superior for preoperative assessment and for identifying the site of obstruction, it may not always identify subtle communications between the duplicated vaginas [11].

Surgical management of OHVIRA syndrome encompasses several techniques, including vaginal septum incision, formal resection, hysteroscopic intervention, laparotomy, and laparoscopy. The definitive treatment of choice is a single-stage resection of the vaginal septum. The primary surgical objective is the complete excision of the septum to establish a permanent, patent outflow tract for menstrual drainage. The anatomical location and thickness of the septum are critical factors in determining the optimal surgical approach. For instance, when the septum exceeds 2 cm in thickness, an abdominoperineal approach is often recommended, as complete vaginal resection can be technically challenging and may result in postoperative vaginal defects [9]. Recently, hysteroscopic (vaginoscopic) incision of the vaginal septum has been introduced as minimally invasive alternative [12]. This approach is particularly advantageous for pediatric and adolescent patients with narrow or immature vaginal canals, offering reduced postoperative pain and preservation of hymenal integrity. While laparoscopy is not routinely indicated for primary for diagnosis and treatment, in may be necessary—potentially including hemi-hysterectomy—in cases involving extrinsic proximal vaginal septum or a serious infectious complication.

## 4. Conclusions

In conclusion, OHVIRA syndrome is a rare Müllerian anomaly that may manifest atypically as recurrent vulvovaginitis during adolescence. Clinicians should consider underlying Müllerian anomalies in adolescent patients with vulvovaginitis that remains refractory to conventional therapy. Prompt diagnosis and appropriate surgical intervention are crucial for symptom resolution and the prevention of long-term sequelae, such as endometriosis and impaired fertility.

## Data Availability

All data used in this study can be obtained by contacting the corresponding author via e-mail at cholida73@naver.com.

## Abbreviations

OHVIRA syndrome, obstructed hemivagina and ipsilateral renal anomalies syndrome; HWW syndrome, Herlyn–Werner–Wunderlich syndrome; AMH, anti-Müllerian hormone.
